# Performance-Cost Trade-Off in Auto-Scaling Mechanisms for Cloud Computing †

**DOI:** 10.3390/s22031221

**Published:** 2022-02-05

**Authors:** Iure Fé, Rubens Matos, Jamilson Dantas, Carlos Melo, Tuan Anh Nguyen, Dugki Min, Eunmi Choi, Francisco Airton Silva, Paulo Romero Martins Maciel

**Affiliations:** 1 Brazilian Army, Third BEC, Picos 64606-000, Brazil; sciure@3bec.eb.mil.br; 2 Coordination of Informatics, Federal Institute of Education, Science and Technology of Sergipe, Lagarto 49400-000, Brazil; rubens.junior@ifs.edu.br; 3 Informatics Center, Federal University of Pernambuco, Recife 50740-560, Brazil; jrd@cin.ufpe.br (J.D.); casm3@cin.ufpe.br (C.M.); prmm@cin.ufpe.br (P.R.M.M.); 4 Konkuk Aerospace Design-Airworthiness Research Institute (KADA), Konkuk University, Seoul 05029, Korea; anhnt2407@konkuk.ac.kr; 5 Department of Computer Science and Engineering, College of Engineering, Konkuk University, Seoul 05029, Korea; 6 School of Software, College of Computer Science, Kookmin University, Seoul 02707, Korea; emchoi@kookmin.ac.kr; 7 Laboratory PASID, Federal University of Piaui, Picos 64600-000, Brazil; faps@ufpi.edu.br

**Keywords:** cloud computing, performance evaluation, cost evaluation, optimization, auto-scaling, stochastic Petri net

## Abstract

Cloud computing has been widely adopted over the years by practitioners and companies with a variety of requirements. With a strong economic appeal, cloud computing makes possible the idea of computing as a utility, in which computing resources can be consumed and paid for with the same convenience as electricity. One of the main characteristics of cloud as a service is elasticity supported by auto-scaling capabilities. The auto-scaling cloud mechanism allows adjusting resources to meet multiple demands dynamically. The elasticity service is best represented in critical web trading and transaction systems that must satisfy a certain service level agreement (SLA), such as maximum response time limits for different types of inbound requests. Nevertheless, existing cloud infrastructures maintained by different cloud enterprises often offer different cloud service costs for equivalent SLAs upon several factors. The factors might be contract types, VM types, auto-scaling configuration parameters, and incoming workload demand. Identifying a combination of parameters that results in SLA compliance directly in the system is often sophisticated, while the manual analysis is prone to errors due to the huge number of possibilities. This paper proposes the modeling of auto-scaling mechanisms in a typical cloud infrastructure using a stochastic Petri net (SPN) and the employment of a well-established adaptive search metaheuristic (GRASP) to discover critical trade-offs between performance and cost in cloud services.The proposed SPN models enable cloud designers to estimate the metrics of cloud services in accordance with each required SLA such as the best configuration, cost, system response time, and throughput.The auto-scaling SPN model was extensively validated with 95% confidence against a real test-bed scenario with 18.000 samples. A case-study of cloud services was used to investigate the viability of this method and to evaluate the adoptability of the proposed auto-scaling model in practice. On the other hand, the proposed optimization algorithm enables the identification of economic system configuration and parameterization to satisfy required SLA and budget constraints. The adoption of the metaheuristic GRASP approach and the modeling of auto-scaling mechanisms in this work can help search for the optimized-quality solution and operational management for cloud services in practice.

## 1. Introduction

Cloud computing is a service-driven computing model whereby an end-user will provide computing resources from a cloud service provider (CSP) in line with an agreed-upon service-level agreement (SLA). The service hosted by the CSP could take many forms, consisting of networking, storage, or computational components. Usually, cloud environments are multilayered. The composition differs depending upon the CSP infrastructure, the application’s use-case, or the particular model used for analysis [1]. Cloud computing provides on-demand access to shared computing resources and services, such as network infrastructure, storage, operating systems, and applications. Such resources and mechanisms can be easily acquired and released with minimal management effort [2]. These features enable administrators to focus only on the business model without worrying about infrastructure details [3]. The experience in acquiring cloud services is often compared to the consumption of public utilities. Two of the key features of cloud computing are the user’s ability to pay only for what they use and deliver resources elastically. The cloud enables reduction costs while meeting the performance requirements of maintaining an application subject to a variable load. Auto-scaling helps keep the trade-off between performance and cost by automatically adjusting the number of resources and the variation of the users’ demand. The user application requires resources that might be seasonal due to variable workload demand in the cloud context. Several concepts and technologies support the cloud computing environment with elasticity provided through the automatic scaling of computing capacity provided by auto-scaling. The main concepts needed to understand the contextualization of this work and the problem will be discussed below.

Virtualization allows a user or process to have the impression of working on a dedicated machine with a complete operating system. Hardware resources can be emulated so that a virtual machine offers computing to the user. Virtual machines are managed and controlled by virtual machine managers (hypervisors) that provide a hardware abstraction for each VM. The ability to create and destroy virtual machines offered by virtualization allows cloud computing to automatically adjust the number of resources for customers [4]. The VMs provisioning tries to match specific hardware characteristics and software requirements of an application.

Most cloud providers offer a set of general-purpose VM classes with generic software and resource configurations. For example, Amazon EC2 supports several families of instance types that are classified according to their use-cases [5], each one with different options of processors, memory, and I/O performance. Instantiating virtual machines in an optimized way may attend peak demand, but the overprovisioned resources might incur high costs on low-demand periods.

Auto-scaling mechanisms: Cloud computing offers on-demand self-service. A customer can access computing resources without requiring human interaction with the service provider [6]. Cloud computing also delivers rapid elasticity, which is the ability to add and release resources according to the demand [6]. Auto-scaling techniques are used to provide an automatic solution for resource allocation [7,8,9,10]. Auto-scaling techniques are usually divided into predictive and reactive. The predictive techniques try to find out the future resource requirements and provide those resources in advance. The reactive ones are based on rules to respond to system changes when they reach predefined thresholds [10,11]. The reactive techniques are the approaches most widely used in commercial systems [9,12]. In Amazon, the application provider can monitor resources or logs and then manually react to predefined alerts. Amazon also enables the user to apply auto-scaling to add a new virtual machine (VM). Additionally, the Amazon Elastic Compute Cloud (EC2) API enables the remote management of the virtual infrastructure to retrieve VMs information and delete and create new instances, among other activities, without the need to directly access the Amazon dashboard [13]. The reactive auto-scaling techniques use at least two parameters: (i) one threshold to instantiate new VMs (i.e., scaling up) when a resource reaches a utilization limit or the system reaches a specific state; and (ii) one threshold of destroying VMs (i.e., scaling down) when there is more capacity than is needed to supply the demand. This action reduces operating costs when there is low demand. The administrator can also determine the amount of VMs to be added or removed when the threshold is reached, which is known as step size [14]. It is worth highlighting that VM instances in public cloud infrastructures are created according to predefined types. VM types with more resources (e.g., CPU cores, memory size) have higher prices. Moreover, there are cost differences for different contracts, which usually is chosen between on-demand or reserved instances. On-demand instances follow the *pay-as-you-go* model, so the cloud customer pays the VM per hour of usage, which might be interrupted anytime. On the other hand, reserved contracts use predetermined times, often intervals of one year. A reserved VM with high computational capacity is usually cheaper than an on-demand VM with the same resources for a full year. A common problem is the wrong selection of parameters for auto-scaling, leading to noncompliance of the SLA or higher costs than necessary [14]. Thus, the creation of models is important for predicting the system performance and cost, considering a given workload demand and auto-scaling settings. In addition, such models may guide the decision-making for the configuration of cloud infrastructure resources and related elasticity mechanisms. An auto-scaling mechanism monitors application metrics against predefined thresholds to add or remove VMs. The proper thresholds definition may not be a simple task [9]. Typically, thresholds definition requires in-depth application knowledge. The main auto-scaling parameters normally include VM types, the quantity of reserved VMs, contracts types (long periods or on-demand), the simultaneous working capacity of each VM, and thresholds for creating and destroying VMs. The processing and instantiation time of VMs is not constant. The influence of these random variables must be predicted, envisioning appropriate system adjustment. The problem tackled in this work is that finding an optimized VM configuration and auto-scaling configuration in this vast possibilities space is time-consuming and extremely complex. Additionally, such optimization can lead to non-SLA compliance. One alternative to mitigate the difficulties mentioned above is to combine stochastic models and optimization algorithms. Therefore, this paper aims to find an optimized trade-off between performance and cost in a cloud computing auto-scaling scenario.

Stochastic Modeling: Petri net is a tool to represent and investigate real or planned information and computing systems with desired levels of detail. Systems are represented by a network composed of a set of places, transitions, tokens, and arcs. Each event takes the network to a new state. For each transition, the model specifies which places will be affected. This characteristic makes the Petri nets an adequate tool for representing distributed systems with concurrency behavior [15]. The original Petri net does not have the notion of time for performance analysis. For this, we will use the stochastic Petri net (SPN), an extension of the Petri net that allows the association of times with the timed transitions of the model [16]. An SPN can be translated to a CTMC, which may then be solved to obtain performance metrics. This is especially useful because building a Markov model may be tedious and error-prone, especially when many states become very large. Marsan et al. proposed GSPN, which is an extension of basic SPN and adopts two types of transitions: timed and immediate [17,18,19]. The timed transitions have delays exponentially distributed, and immediate transitions fire in zero time. Currently, the acronym SPN is often used to represent the stochastic Petri nets subfamily of models derived from the original SPN model [20].

Due to the memory-less property, SPN models with numbers of places and finite transitions are isomorphic to CTMC models, so they can be translated into CTMCs and solved numerically to obtain performance metrics. This solution method provides accurate results, but it cannot always be applied. One of the limitations is that the distribution associated with timed transitions must be exponential, which can be bypassed through moment matching techniques, creating new transitions and places. However, this solution can contribute to the other restriction, which is the explosion of the CTMC state space generated by the SPN, making the time for computing the metrics prohibitive [21]. On the other hand, simulation techniques can also obtain the metrics, an alternative when some of the above restrictions are not satisfied. For example, simulation methods allow other distributions for the timed transitions (usually represented as a gray rectangle) and for not needing to generate the CTMC. However, in an SPN model that adequately represents a system, the results of the metrics obtained by numerical analysis may be more accurate than those obtained by simulation (considering the simulation and numerical analysis of the same SPN model that faithfully represents the system). This happens because in the simulation, the results are presented within a confidence interval; in contrast, the values obtained by numerical analysis are punctual [21]. Thus, stochastic modeling is a suitable and widespread formal method for performance evaluation in concurrent systems, synchronization, and communication mechanisms.

Stochastic modeling is appropriate for both deterministic and nondeterministic events. Stochastic Petri nets (SPNs) are special cases of stochastic models [22,23,24,25,26,27,28,29,30,31,32,33]. SPNs enable setting up of state equations, algebraic equations, and other mathematical models governing the behavior of systems. *GRASP (greedy randomized adaptive search procedure)* is one of the most effective techniques for combinatorial optimization problems [34]. GRASP combines local search procedures with an iterative, semi-greedy, and random strategy. GRASP creates a solution based on infinite possibilities. Therefore, this paper proposes to combine stochastic modeling with metaheuristics of the GRASP method. Our strategy aims to identify a way to find solutions with lower costs that satisfy the SLA constraints and present a viable solution. Other works in the literature also looked for methods of finding optimal resource allocation, as in [35,36,37], where the authors sought to use cloud resources to provide adequate performance in storing files at a lower cost. Other works also sought to reduce the cost by maintaining the SLA in database distribution environments supported by cloud computing [38].

Literature review: A common objective in previous work is to obtain the maximum resources utilization of a cloud-hosted application through auto-scaling [39], although a common mistake in many cloud projects comes from misconfiguration that often results in overprovisioning [40]. Reducing cost also has been explored; Aslanpour et al. [41], for example, executed scale-down commands via a selection of virtual machines, decreasing the billing period in order to maximize the cost-efficiency. Other works use models to manage the trade-off between cost and performance together [42]. However, none of these related works have explored the following characteristics in conjunction: architecture planning, formal optimization, VM types parameterization, types of contracts observation, and, finally, stochastic modeling representation.

The contributions of the paper are summarized as follows:

- We propose an SPN model to capture sophisticated auto-scaling mechanisms in a typical cloud computing system.The model addresses the application process, the VM instantiation mechanism, and the VM termination mechanism. The model has the main purpose of calculating the mean response time, throughput, and cost of the VMs in different auto-scaling configurations. The proposed model is an extension of our previous validated auto-scaling model for performance and cost evaluation [43].
- We adopted the GRASP optimization algorithm to investigate the most suitable model parameters. We calibrate system parameters to achieve a configuration that respects service-level agreements and optimizes the cost.The space of possible solutions includes both scenarios—public and private cloud systems. It was possible to find an adequate trade-off between performance and cost regarding the auto-scaling mechanism by combining the model and the optimization algorithm.
- We adopted the proposed methodology in a practical case study of cloud services to demonstrate the feasibility of the proposed model and optimization algorithm.We explored feasible solutions for a public cloud aiming to identify an optimized configuration in video transcoding systems.

The remaining of this paper is organized as follows: Section 2 summarizes the more closely related works to our proposal. Section 3 describes the architecture taken into account by the modeling and experiment activities. Section 4 presents the SPN model and how anyone can use it. Finally, Section 4.3 gives the details about the optimization algorithm and how it improved the SPN model results. Section 5 illustrates how to use the proposed model in a very practical way. Finally, Section 6 outlines some conclusions and future work.

## 2. Related Works

This section presents related works with approaches similar to our proposal. These works deal with different auto-scaling metrics and parameters. The most important goal sought in most papers is to develop a technique that may achieve the SLA with the lowest possible cost. Another common objective is to obtain the maximum resources utilization of a cloud-hosted application through auto-scaling. For this, Wei et al. [39] proposed a Q-learning-based self-adaptive renting plan generation approach to help SaaS providers make efficient IaaS facilities adjustment decisions dynamically to help SaaS providers make optimal resource allocation decisions in a dynamic and stochastic cloud environment. Furthermore, they considered different VM pricing mechanisms in their model, including on-demand patterns and reserved patterns. We consider different contract types and the ability of VMs to present more cost-effective configurations for the same SLA. Our optimization approach can predict the optimized cost for a variation of workload and configuration by using the SPN model combined with optimization.

Aslanpour et al. [44] proposed an executor to reduce cost, which shows the importance and effectiveness of monitoring, analysis, planning, and execution. Their solution executes scale-down commands via an aware selection of surplus virtual machines, decreasing the billing period to maximize cost efficiency. Furthermore, this approach shows that the proposed executor reduces the cost of renting virtual machines while improving the application’s final service-level agreement. Our work considers more configuration elements, such as the number of simultaneous jobs and VMs instantiated at a time, which brings a greater possibility of finding configurations that meet the SLA at a lower cost. We also present a method that considers the cost as a function of the SLA, not just cost reduction.

Huang et al. [45] used the on-demand features of cloud computing to provide a database virtualization solution that meets SLA performance requirements. They applied the auto-scaling mechanism on the route server in the database system. The results demonstrated the advantage of using auto-scaling in database systems. An algorithm was also created to determine how many VMs to add to the system. However, this work does not present the total VMs used cost. Unlike our work, it does not present a cost reduction optimization methodology for dynamic resource allocation, proposing this approach in future works.

Other works use models to manage the trade-off between cost and performance, such as Shahidinejad et al. [42], who proposed an elastic controller based on colored Petri nets to manage cloud infrastructures automatically. They evaluated the efficiency of the proposed elastic controller focusing on average response time and CPU utilization. Shahidinejad et al. [42] presented a useful application of modeling a cloud system to improve performance and maintain the SLA. However, unlike our work, their work did not consider cost as an optimization metric.

Huang et al. [46] proposed a queuing model M/M/C based on this queuing model. They used heuristic algorithms and dynamic programming methods to design virtual machine (VM) auto-scaling strategies. The proposed model and scaling algorithms make web applications and use the least resources, improving resource utilization and minimizing deployment costs. Evangelidis et al. [47] also proposed a performance model with formal verification that resulted in rule-based auto-scaling policies. They ensured the usefulness and efficiency of their technique through validation in cloud providers. Their experimental results show that the modeling process and the model itself can effectively provide the necessary formal understanding to cloud application owners. The configuration understanding of their auto-scaling policies can consequently help them specify an auto-scaling policy that could minimize QoS violations. Unlike our approach, these last two works did not consider different types of VMs, which, in our results, was shown to be a configuration element that can reduce cost depending on the SLA.

Schuler et al. [48] deals with an application that uses reinforcement learning to achieve high quality of service dealing with a variable workload and complex infrastructure characteristics, such as throughput and latency. His results demonstrate that with a low number of iterations, the proposed learning model increases the performance of a system compared to the standard auto-scaling configuration. Our work presents a different approach by addressing beyond latency, adding mean response time to cost, and an optimization algorithm that handles SLA constraints to present the lowest cost configuration.

As presented in this work, Bauer et al. [49] used a similar approach to configure a threshold, demonstrating that an auto-scaler that leverages information about service demands significantly outperforms auto-scalers solely based on CPU utilization measurements. This is shown by testing two approaches in three different scenarios. Their results show that the service demand-based auto scaler outperforms the CPU utilization-based one in all scenarios. Unlike what was presented by Bauer et al. [49], we were more comprehensive in using more elasticity configuration parameters and in the use of an optimization algorithm to obtain an optimized configuration. Table 1 presents an overview of the related works of this paper. The studies address auto-scaling techniques focusing on improving performance or cost. Studies from 2016 to 2020 are listed. Five aspects were observed over these studies: metric, architecture planning metric, formal optimization, different types of VM, and different types of contracts.

Architectural planning—This is an important feature to predict the financial impact of fulfilling a given SLA. Although many works focus on identifying a good auto-scaling mechanism, few allow identifying the impact of workload variation on the expected total cost of a cloud system. The works aligned with this feature allow planning through the use of their models but still require an elaboration of a simulation model [44,47]. Our approach can easily be adapted in different use cases, using the SPN model and optimization algorithmic.

Measured metrics—Response time is often used to identify web application performance SLA. High response times can result in useless applications and are associated with a lack of computing resources. Cost is often presented as a trade-off to performance, and a high cost can result from high demand or incorrect configuration. Similar to most other works, we consider response time and cost, but we deal with throughput that could be a significant metric in some scenarios.

Formal optimization—This describes whether any optimization method was used. The use of optimization implies using some technique that allows investigating the solutions space with the methodology of the work. It enables to lead to a configuration that minimizes or maximizes a metric of interest. Using formal configuration optimization methods can help to identify good solutions to the trade-off between performance and cost. Bauer et al. [49] compared an optimal algorithmic that uses CPU and service demand based on service demand law as a threshold. In turn, Wei et al. [39] used a Q-learning-based self-adaptive renting plan generation algorithm to find optimal resource allocation. In this work, GRASP assisted in finding a possible combination of the different parameters and presenting a possible configuration to be used in the auto-scaling configuration that respects the SLA and, at the same time, reduces the total cost. The use of a metaheuristic to search for the solution allows a greater guarantee of an adequate solution for practical uses by the search methodology carried out in the solution space. Using GRASP with the SPN model allows not only finding the optimized solution but also identifying how the system should be configured in different scenarios of workload variation, SLA, or the feasibility of other VM types in the system.

Different types of VMs—This refers to solutions that take into account different types of VMs, with different capacities and costs, which produce solutions with one more cost-reduction factor in a configuration that respects a given SLA.

Different types of contracts—These are related to works that take into account different types of virtual machine contracts. In this work, the use of reserved and on-demand VMs was considered, and the combination of both contracts is a possible solution for cost reduction.

Our work proposes a methodology that combines formal modeling with an optimization algorithm. Our approach allows fine-tuning of several auto-calling parameters, VM types, and contract types. We consider stochastic cloud elements such as the different response times for each type of VM as the different instantiation times of different VMs. The approach allows the prediction of the best configuration, cost, system response time, and throughput for each required SLA, thus helping both reduce costs while maintaining the SLA and studying several hypothetical scenarios.

## 3. An Auto-Scaling Cloud Architecture

**Figure 1:**
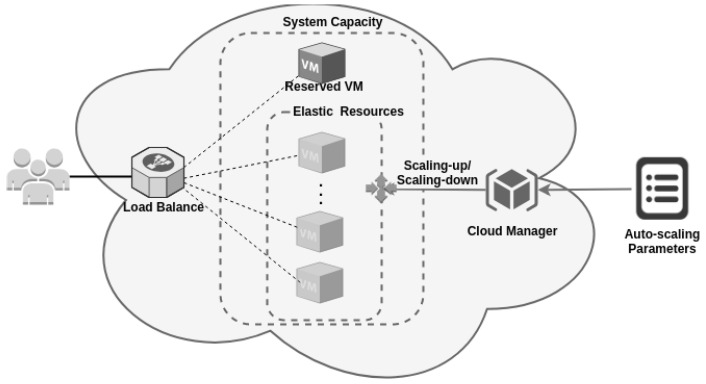
A typical architecture of auto-scaling cloud systems.

Clients send requests to a load balancer in a typical cloud web application that distributes requests to VMs. These VMs can be of two types: reserved and on-demand. If there are no available VMs, the requests can be stacked, waiting for an available VM. When a VM becomes available, it receives a request in the queue by the first-come-first-served (FCFS) policy. The system employs reactive auto-scaling by monitoring the number of jobs in the system queue and using that information in the scaling-up threshold. Reactive auto-scaling can also be performed by tools available in most public cloud providers, such as Amazon CloudWatch [7,9,13]. Figure 1 depicts a general view of flexible systems architecture in the cloud. Dark-blue blocks represent a reserved instance, and light-blue blocks represent the on-demand instances. The load balancer is responsible for receiving requests and forwarding them to the VMs that perform the service. The auto-scaling parameters are responsible for determining the moment of instantiation and destruction of the elastic VMs.

Several VM configuration factors determine throughput, mean response time, and cost of the system. [43] presented an some prices on Amazon for distinct VM types and their resources characteristics, which are reflected in the meantime. The main four types of instances include I: t2.micro (1 core, 1 GB RAM); II: t2.small (1 core, 2 GB RAM); III: t2.medium (2 cores, 4 GB RAM); IV: t2.large (2 cores, 8 GB RAM). Regardless it is possible to use other VM types and prices doing respective price changes. VMs with more vCPUs (virtual processors) respond faster and have higher costs than VMs with fewer resources. The types of VMs might also influence the time to boot the operating system of the new on-demand instances. There is also the time for starting the software stack responsible for receiving requests.This time for initialization activities reflects a delay in responding to the requests while the machine is not fully operational. Therefore, the amount of reserved VMs might also affect metrics of interest.

The greater their amount, the greater the system’s response capability, avoiding the delay of the instantiation on demand. However, this contract mode will charge even in the load absence.

## 4. Proposed SPN Modeling and Optimization Algorithm

### 4.1. System Model

As shown in Section 3, proper configuration of public cloud-based systems requires fine-tuning of various configuration parameters at the service provider. The modeling of this type of system must take all these parameters into account. Some of the parameters are directly related to cloud elasticity, such as scaling up or scaling down threshold or number of VMs allocated to each threshold. Other parameters are derived from the contract with the provider.

**Figure 2:**
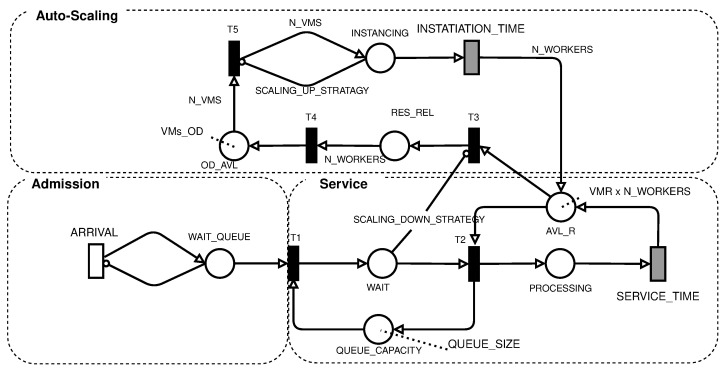
SPN model for cloud-based system.

The choice of the previous configurations also has effects on the modeling of the system. Setting the VM type changes the expected average times for processing a request and affects the time to instantiate a new VM dynamically. Processing time is also affected by the number of concurrent jobs running on the system. The model in Figure 2 took all these factors into account. The description of the places and transitions of the model can be seen in Table 2. In comparison, the attributes of the immediate and timed transitions can be seen in Table 3. Table 2 presents the description of places and transitions of the system configuration. In this table are both the values directly defined by the user, such as the THR_SCALING_UP, and the time values that are a consequence of choosing the VM type, which is SERVICE_TIME and INSTANTIATION_TIME. Table 4 has model variables that represent system characteristics such as workload (represented by the ARRIVAL variable) and the number of jobs waiting in the queue. Additionally, the characteristics of the immediate and timed transitions are listed in Table 3.

The values of the timed transitions must be obtained for each VM type. The average processing time and instantiation time must be obtained for each type of application by the system administrator and are obtained through controlled measurements of requests in the system.

**Figure 3:**
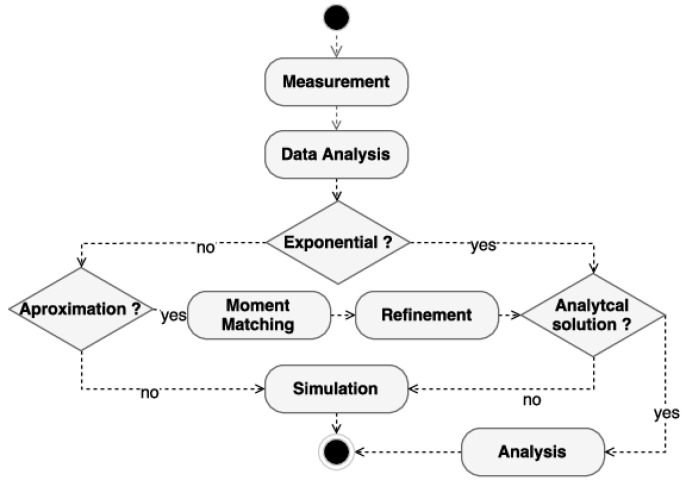
Evaluation method selection.

The mean time values obtained must be evaluated as to the type of distribution, which will be used to choose the model evaluation method. A methodology for identifying the type of assessment can be seen in Figure 3. The data collected for these average times must be submitted to data analysis to identify the possibility of using analytical solutions or if the model will only be run by simulation. The model can be run looking for analytical solutions if the model has exponentially distributed time transitions and does not suffer from a state-space explosion, which can occur with large systems with many VMs or many jobs per VM. The state-space explosion can generate a model with high execution time, making its use unfeasible for optimization, which will need to run the model with different values countless times.

If it is not exponential, the feasibility of using moment matching to use sets of exponentials to represent another distribution can be verified [22]. The sub-model that represents the distribution must refine the original model and generate an alternative model composed only of exponential distributions. This alternative must also be the target of verifying the feasibility of use regarding the explosion of state space. Suppose it is not possible to evaluate the model composed of exponential transitions. In that case, it is still possible to run the model by simulation, which, although does not present punctual results as the numerical analysis, presents results within good enough confidence intervals for practical applications. Therefore, modeling the model with SPN can be suitable for several different configurations, and the chosen evaluation method should be carefully checked.

The proposed SPN model of cloud-based systems with auto-scaling is shown in Figure 2, being composed of three subnetworks: admission, service, and auto-scaling. The admission subnet generates the workload and represents the time between requests. It is composed of an ARRIVAL transition, which is a single server transition and has the expected mean arrival time value, and the **WAIT_QUEUE** place, which represents a request that is ready to enter the system.

The service subnet is responsible for receiving the requests from the admission subnet and forwarding the request to an available VM that will carry out the processing. This subnet starts with firing the T1 transition, which occurs whenever there is capacity in the **QUEUE_CAPACITY** load balancer. The FCFS load balancer starts with the number of tokens equal to **QUEUE_SIZE**, which is the maximum capacity of the system. This capacity is reduced as new requests enter the system and are waiting in the **WAIT** place. Requests in **WAIT** are used to check the system’s occupancy level, and you can increase or decrease the number of VMs by scaling up AND scaling down strategies, respectively.

The **T2** transition fires whenever there are requests waiting and computational resources available; that is, there are tokens in **AVL_R**. The initial number of tokens in **ACL_R** is equal to the amount of reserved VMs multiplied by the amount of concurrent work that each of these virtual machines can perform (**VMR** x **N_WORKERS**). After firing T2, the request will be processed in place **PROCESSING**. The number of tokens in **PROCESSING** represents the number of requests being processed simultaneously. The processing time is given by the **SERVICE_TIME** transition, which represents the processing time allocated to the request.

The **SERVICE_TIME** timed transition obeys infinite server semantics. Each request is processed independently of the other. The time required to process a request depends on the amount of concurrent work. Usually, processing simultaneous requests in a single VM can take more time per request than just one request. Therefore, the time used in this request should be measured under similar workload conditions.

The number of tokens in **AVL_R** and **PROCESSING** is changed by the auto-scaling subnet, never being less than the initial value. The scaling up is performed whenever the **SCALING_UP_STRATAGY** condition is satisfied. The change in the model is performed by firing the **T5** transition. The amount of VMs added to each scaling up of the threshold of the condition is given by the variable **N_VMS** and is conditioned by the capacity of available resources in place **OD_AVL**. The capacity in **OD_AVL** in public clouds can be considered unlimited due to the large capacity of large cloud providers, whereas in private clouds, the capacity depends on the installed infrastructure.

The firing of **T5** depends on the **SCALING_UP_STRATAGY** (1) condition, which defines the multiplicity of the inhibiting arc. This condition enables the T5 transition whenever the number of tokens in the INSTANCING place is less than the value resulting from this condition. The condition value (1) checks if the number of requests waiting (**WAIT**) is greater than the threshold **THR_SCALING_UP** multiplied by the number of VMs in the instantiation process and those already running (**INSTANCING**, **AVL_R**, and **PROCESSING**). If this condition is true, the arc multiplicity will become the number of VMs in instantiation added to 1, enabling **T5**. On the other hand, the multiplicity of the inhibiting arc is zero, disabling the **T5** transition.

(1) $$\begin{array}{cc} IF(\#WAIT & >=((\#INSTANCING+\\ & ((\#AVL\_R+\#PROCESSING)/N\_WORKERS))\times THR\_SCALING\_UP)):\\ & \left(\#INSTANCING+1\right)\\ ELSE & \\ & \left(0\right) \end{array}$$

The number of VMs to be instantiated is equal to the **N_VMS** parameter; these tokens are deposited in the **INSTANCING** place and they wait for the time given by the infinite server **INSTATIATION_TIME** transition. This time depends on the type of VM used and also on the cloud environment used. After firing **INSTATIATION_TIME** for each VM, the **N_WORKERS** capacity is deposited in **ACL_R**, increasing the number of resources available to process the requests. It is important to note that the scaling up mechanism is dependent on the **THR_SCALING_UP** variable, which is defined by the user in both the cloud management system and the model.

The mechanism for destroying VMs on demand is controlled by condition (2) in the **SCALING_DOWN_STRATEGY** arc. It changes the enabling of the **T3** transition. This mechanism aims to save resources when the current amount of VMs on demand is no longer sufficient. Condition (2) evaluates if there was the instantiation of VMs on demand. If any VMs were instantiated, then the arc will compare the number of requests in WAIT with the total running capacity, **ACL_R**, **PROCESSING**, and **RELEASE_RESOURCE** multiplied by the value of **THR_SCALING_DOWN**. If **T3** fires, it will gather the resources of the VM on-demand in **RELEASE_RESOURCE**, which represents the wait for the completion of the work being carried out by the VM. When the scaling down on-demand VM ends its processes, **T4** is enabled, which returns the on-demand resources leased from the cloud.

(2) $$\begin{array}{cc} IF(VM\_OD>\#OD\_AVAL):\\ & (\left(\left(\left(\#ALV\_R+\#PROCESSING+\#RES\_REL\right)/N_{W}ORKERS\right)-1\right)\\ & \times THR\_SCALING\_DOWN)\\ ELSE & \\ & \left(0\right) \end{array}$$

### 4.2. Model Metrics

The purpose of the model is to generate metrics of interest that allow evaluating the performance and cost of a certain configuration of a cloud system. We generate metrics for throughput, average response time, and cost. The throughput is calculated by the expected number of tokens in **PROCESSING** multiplied by the service time rate, as presented in Equation (3). For an Erlang distribution, throughput can be calculated as presented in Equation (4).

(3) $$\begin{array}{c} TP_{Exponential}=\left(\sum_{i=1}^{n}P\left(m\left(PROCESSING\right)=i\right)\times i\right)\times \frac{1.0}{SERVICE\_TIME} \end{array}$$

(4) $$\begin{array}{c} TP_{Erlang}=\left(\sum_{i=1}^{n}P\left(m\left(PROCESSING\right)=i\right)\times i\right)\times \frac{1.0}{TSERVICE\_TIME\times KSERVICE\_TIME} \end{array}$$

The average response time of the system can be calculated using Little’s law. This metric takes into account the number of jobs in the system and the inter-job arrival rate (**ARRIVAL**). For this model, the application of Little’s law results in Equation (5), in which the effective arrival rate was considered, that is, disregarding the fraction of the arrival rate that may have been discarded.

(5) $$\begin{array}{c} M_{RT}=\frac{(\left(\sum_{i=1}^{n}P\left(m\left(WAIT\right)=i\right)\times i\right)+\left(\sum_{i=1}^{n}P\left(m\left(PROCESSING\right)=i\right)\times i\right))\times ARRIVAL}{1-P((WAIT\_QUEUE=1)\wedge (QUEUE\_CAPACITY=0)\wedge (AVL\_R=0))} \end{array}$$

Finally, we present the infrastructure cost, which depends on the used portion of on-demand VMs used (ON_DEMAND_USE) in Equation (6). The sum of reserved and on-demand VMs can be obtained by Equation (7). This equation considers the cost over some time T (in years). The annual cost of reserved and on-demand VMs are, respectively, $VM_{C\_Res}$ and $VM_{C\_Ond}$. Table 5 presents the model metrics as they should be used in the Mercury tool.

The model and some metrics presented in this section are adaptations of those found in our previous work [43]. In this work, we use the model for use in systems with response times that are not exclusively exponential. We adopt Erlang time distribution in our use case and present Equation (4) to calculate the flow rate in systems with this distribution. In addition, we have added a methodology and a flowchart to guide the use of the model for larger systems with multiple requests or a large number of resources acting simultaneously. This methodology also contemplates the model execution and customization options with phase-type distributions when the transition times are not exponentially distributed. A more sophisticated validation was also performed, which includes a more complex workload and simulation, as will be discussed in detail in Section 5.1.

(6) $$\begin{array}{c} ON\_DEMAND\_USE=VM\_OD_{initial}-\sum_{i=1}^{n}P\left(m\left(OD\_AVL\right)=i\right)\times i \end{array}$$

(7) $$\begin{array}{c} Cost\left(T\right)=ON\_DEMAND\_USE\times T\times VM_{C\_Ond}+VM_{R}\times T\times VM_{C\_Res} \end{array}$$

The model and metrics presented in this section allow the systems manager to assess the impact on the performance and cost of a system hosted in the cloud from the variation of elasticity configuration parameters, contracts, and types of VM used.

### 4.3. Optimization Algorithm

The models have several parameters and possibilities of combinations of parameter values. These combinations present different values for the metrics of the model. The model explores the space solutions through the individual variation of each value of the parameters. Searching for an optimized configuration is an exhaustive task and, in many cases, impractical. Therefore, we use optimization mechanisms. This section explores the space of possible solutions for the public and private cloud systems. We seek to identify the values configured in each parameter to achieve a configuration that respects the SLA and optimizes the cost. This process uses the GRASP optimization algorithm, which was adapted to search for the model’s variables and adopt the cost metric of the model as the objective function.

The optimization algorithm used receives as input the model, the parameters that must be configured, the ranges of variation of each parameter, the constraints of performance metrics (throughput and average minimum response time), the workload that the system is subjected to, and the values associated with the chosen cloud. After that, the algorithm generates solutions from the set of possibilities that optimize the cost.

This work adopts GRASP as the metaheuristic to search for good-quality solutions. This metaheuristic was presented in 1989 in [50]. GRASP is an iterative, semi-greedy metaheuristic also possessing randomness. The GRASP implementation was developed in three algorithms. Algorithm 1 is a generic high-level representation of GRASP that follows the strategy defined in [50]. Algorithms 2 and 3 are applications of this work to implement cost optimization of the SPN model previously presented.

GRASP creates a process capable of escaping from local minimum and performing a robust search in the solution space [51]. This type of optimization method produces good-quality solutions for difficult combinatorial optimization problems.

In GRASP, each iteration consists of two phases: the construction and the local search. The construction creates a solution at random; if the solution is not acceptable, a repair may be applied. During the local search, the neighborhood of the generated solution will be investigated until finding a local minimum.

The high-level GRASP is presented in pseudocode in Algorithm 1. This algorithm receives, as parameters, the number of iterations (max_iteration) and the seed (seed) for random generation. Furthermore, the first solution is defined as infinite at the algorithm initialization, ensuring that the first solution replaces the first one. Initially, random greedy solutions are constructed and are refined by the local search. We want, in this study, to minimize the cost; thus, if the found solution has the lowest cost than the previous lowest best_solution, it will be replaced in line 5 of Algorithm [50,52].
**Algorithm 1:** Greedy randomized adaptive search procedure—GRASP.
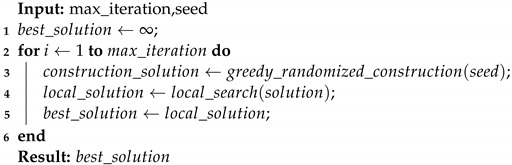


The first phase consists of random construction of greedy feasible solutions (line 3), where every possible element belonging to each parameter will be evaluated from the model with an initial configuration. The best elements concerning cost will be inserted in the restricted candidates’ list (RCL). Later, the RCL elements will be randomly chosen to make a solution. According to the problem, the choice of RCL elements is defined by the greediness parameter, which is set to a value between 0 and 1. The choice of value 1 means “pure greedy” algorithm because the algorithm will always choose the best element of the RCL. On the other hand, 0 means “purely random”. In our study, the greediness parameter is 0.8, which allows a random element to be chosen from the 20% top-tier elements at the RCL [50,52].

The solution generated in the construction will not necessarily be optimal, even locally. The local search (line 4) phase searches for local minimum interactively. It successively changes the previous solution for a lower cost local. The speed and effectiveness of local search depend on several aspects, such as the nature of the problem, the neighborhood structure, and the search technique. This phase can greatly benefit from the quality of the solution found in the construction phase [51].

Several techniques are used in the local search, such as the variable neighborhood search (VNS) and its variation (VND), which have been applied in several studies in the literature [51,52,53,54]. VNS is based on the principle of systematically exploring multiple neighborhoods combined with a disturbance movement (known as *shaking*) to escape from great locations. VND is a variation of VNS, in which the shaking phase is excluded and is usually deterministic. Both approaches take advantage of the search in more neighborhoods up to a maximum number of searches *k*. We use VND in local searches in this work.

We use Algorithm 1 for optimization; it has two phases, construction and local search. The construction phase should generate a good semi-greasy solution, which, applied to the model, selects a value within a finite range of elements for each parameter. These elements form a valid configuration that meets the SLA for which it minimizes the cost and respects the constraints.

Our application of the construction phase in the model can be seen in Algorithm 2. It receives as input the stochastic model, the parameters with possible internal values (example: number of reserved VMs, which can vary from 1 to 30; or VM types that can be t2.micro, t2.large, each with its service and instantiation time for the load of expected work, etc.), α that will determine the size of the candidate restricted list (RCL), β which will be used to increase the variability of solutions [55], the expected workload for the system, and the SLA of mean response time and throughput.
**Algorithm 2:** Greedy randomized construction for SPN model.
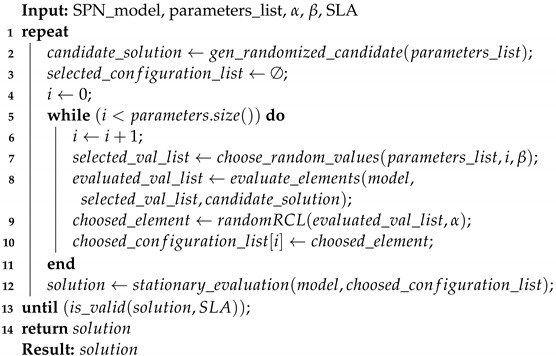


Line 2 generates a candidate solution from the random choice of all possible values for each parameter. The semi-greedy method will select the parameters from line 5 to line 11; the variable *i* will define which parameter will be chosen in the iteration. Line 7 will randomly select part of the possible values for the *i* parameter; if β is 1, all possible values will be chosen, and if it is 0.5, half of the values, with a minimum of 1. This strategy increases the variability of the solutions generated in the construction phase [55]. choose_random_values must also select only the possible values for the parameter; in the case of the public cloud, the value of the threshold of destruction must not be greater than or equal to the instantiation threshold.

After obtaining the values of the *i* parameter to be tested, line 8 will replace each in the candidate solution and evaluate the model, obtaining the cost variation from each value. Next, the parameters are ordered from the lowest to the greater incremental cost and they return a list with the parameters ordered by the cost variation. It is important to note that the model evaluation is performed through the Mercury API call [56] and the solution given by simulation.

The *ramdomRCL* function on line 9 receives the list with the values and costs sorted from lowest to highest and then selects a value randomly within the candidate restricted list (RCL). The RCL is composed of the best in the evaluated_val_list list. Its size is given by α, where “1” denotes “for all elements” and “0” for “only 1”. It is important to note that this is the main random component of GRASP to avoid local minimum. The selected element of the RCL will be used as the value for the *i* parameter of the solution (line 10).

This process will be repeated for all parameters until the solution is composed. The results are inserted into the model and simulated by the Mercury API, where the metrics throughput, average response time, and cost will be generated. The feasibility of the configuration found in the previous steps is verified by the function of line 13, in which it is verified if the presented solution presents metrics to the minimum required SLA. If not, the initial solution is discarded, and another one will be generated.

Local search VND will use the local search and will change the search center to each iteration. This proposal aims to improve the quality of solutions while changing a point with lower local costs. Algorithm 3 presents this strategy applied to the model. It receives the model; the solution of the construction phase; γ, which will define the range of variation; and the maximum number of searches within the local search.

Lines 1 and 2 start the variables used in the model in the same way as the simple local search. However, this algorithm’s maximum execution will not be determined by the maximum number of iterations other than the simple local search. Instead, the maximum amount of execution is determined by the maximum amount of no improvements, given by the variable max_non_improvement. The iteration control variable *i* will be restarted with each improvement and also restarting a new neighborhood.

Line 4 will perform a simple local search from the neighborhood of minor_solution; if it finds a viable new solution that minimizes the solution (condition of line 5), the new solution will be the minimum so that the local search neighborhood will be the new solution and will also restart the iteration variable (line 7). If the solution does not improve, a new iteration will be performed and a new local search will be performed. Note that the variability of solutions that do not find improvement is due to the random aspect of the local_search function. Another important aspect is the successive changes of neighborhoods in the conditions of improvement.
**Algorithm 3:** Local search VND.
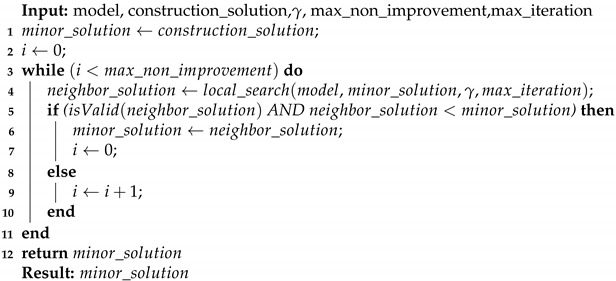


The presented algorithms allow the configuration of the number of iterations parameters in general (Algorithm 1) and in the local search (Algorithm 3). This parameter will define the quality of the solution found and the time to find this value. The more iterations, the longer it will take to execute and will tend to present solutions with lower cost. It is important to note that the execution of this optimization should only be performed with a change in the SLA or system characteristics, such as response time or average VM instantiation time.

## 5. Model Validation and Case Studies

### 5.1. Model Validation

This section presents the results and the methodology used to validate the model presented in Section 4.1. We expose the model and system to a variable workload with exponentially distributed intervals between requests. The workload chosen was sufficient to exhaust the expected capacity of the reserved VMs and exceed the scaling up and scaling down thresholds. The scenarios were planned with the objective that the model and the system dynamically create and destroy VMs while executing the requests. Validation was performed with a 95% confidence interval. We use a private cloud environment for the experiments, but we use a VM with features equivalent to the one in Amazon’s cloud environment. We also use the AWS Elastic Computing API so that the system has the auto-scaling behavior and functionality equivalent to those found in a public cloud environment [13].

A. Testbed architecture: The infrastructure used to run the application and obtain the metrics was composed of two servers with Xeon CPU E3-1220V3, 32 GB of RAM memory, and two gigabit ethernet network interfaces. The servers used Xen Server hypervisors and were managed by the Cloud Stack private cloud environment, which was installed on another server. Two storage servers were also used together to store the results of the request processing. The storage servers used have 4 gigabit ethernet network interfaces and three disks configured in RAID 5.

We used a private switch with 16 gigabit ethernet ports and 32 Gbps maximum capacity. We installed the cloud manager in the server with Core i7 CPU, 4 × 3.40 GHz, 8 MB Cache, 4 GB RAM, 1 gigabit ethernet NIC. The load balance was installed in a VM type **t2.medium**.

The validation was conducted by a web-based video transcoding system that uses the FFMPEG software to process various formats for MP4. Two scenarios were used to validate the model. Scenario I has several conversion requests for the same video, with a duration of 00:04:55, frame sizes (640 × 356), 25 frames per second, and a 200 kbps rate. Scenario II comprises ten videos of different lengths, so that the average duration of their sum is equal to the average length of YouTube videos, 210 s, as described in [57], all with frame sizes (640 × 360), 48 frames per second, and 448 kbps rate. It is important to emphasize that in each scenario, 9.000 requisitions have been met, so the validation of the model comprises 18.000 requisitions.

For both scenarios, we defined parameters used in the system and the SPN model. The type of VM used for video transcoding was **t2.small**; the maximum amount of elastic VMs was three; only one reserved VM was used. The value of the instantiation threshold for on-demand VMs was four. The value of the threshold for destroying on-demand VMs was two. The **N_VMS** and number of simultaneous conversions by VM (**N_WORKERS**) was one.

Unlike our previous work, we present a more comprehensive validation containing videos of different sizes following the size of videos on Youtube, therefore, a more realistic scenario demonstrating the validity of our approach with an expected workload with characteristics similar to those found in large platforms. Furthermore, different from that presented above, we present the validation of our model in a stationary simulation scenario, which is more suitable for use in large systems with large workloads and several resources. The proposal enables us a reduction of time to find solutions. Therefore, we can explore the solution space by repeated execution to find the optimized solution with an optimization algorithm, as required by the algorithms presented in Section 4.3.

B. Experimental results and validation: The mentioned configuration parameters are sufficient to set up the system, but the model needs two other important input values: the service time for the case when **N_WORKERS = 1**; and the VM instantiation time, including their statistical distributions. In order to identify the duration and distribution of transcoding video in VMs of the type **t2.small**, we employed the Jmeter testing tool to generate workload to our private cloud system. We configured Jmeter to send 60 conversion jobs with a delay of 1 min after the end of each transcoding so that the value obtained for **service time** would not have any influence from queuing on the server side. In the case of videos with different lengths, they were randomly selected during the execution of the experiment in Jmeter. Table 6 presents the information for the probability distribution of the transcoding time in both scenarios, 1 and 2. According to the Anderson–Darling and Kolmogorov–Smirnov methods with 95% confidence, there is no evidence to refute that an Erlang distribution in both scenarios can represent the service time distribution.

The next parameter to be measured in the test-bed cloud system was the VM instantiation time. This time value depends on the VM type. Table 7 presents results for tests with a t2.small VM. These values were measured by sending 60 EC2 requests for creating a new t2.small VM instances. The Anderson–Darling and Kolmogorov–Smirnov methods did not indicate any evidence to refute that the instantiation time follows an Erlang distribution, with 95% confidence.

**Figure 4:**
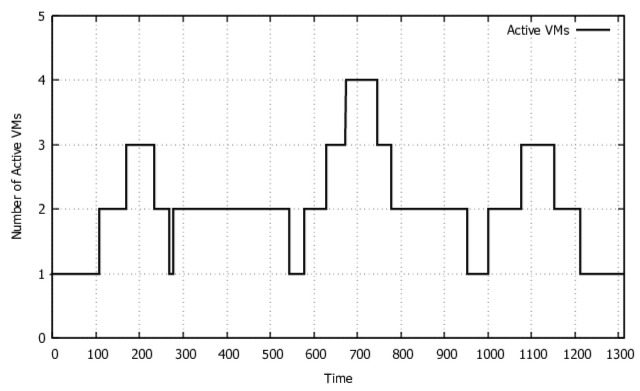
Variation of the number of VMs.

After obtaining the necessary input data for the model, we carried out the validation three times between arrivals of requests for both model and system. Scenario 1 employed inter-arrival times exponentially distributed with 35, 20, and 10 s. These rates between requests are adequate to cause auto-scaling execution (which changes the number of elastic VMs according to the load variation) with the configuration defined above. Both scaling-up and scaling-down execution are part of one of the system executions in Figure 4, and we can observe the number of VMs changing during transcoding execution. Jmeter carried out 30 runs for each inter-arrival time. In each run, 100 videos were converted. Therefore, the results summarize the behavior of the system, considering a total of 9000 video conversions. The throughput and average usage of elastic VMs were measured during these validation experiments.

**Figure 5:**
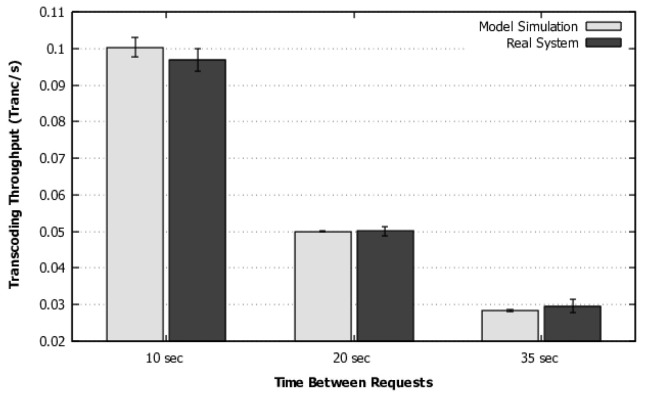
Scenario 1—Validation of throughput.

The model was solved using stationary simulation (to avoid state-space explosion), considering the mentioned Erlang distributions for transcoding and instantiation. Figure 5 shows the comparison of throughput results measured in the system and the model solved via simulation. The model results were consistent with the system results for the three cases of time between requests, considering the 95% confidence intervals that overlap from one to the other.

**Figure 6:**
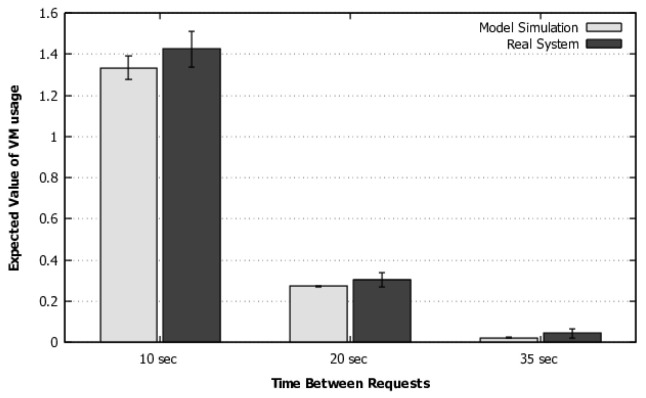
Scenario 1—Validation of elastic VM usage.

Figure 6 depicts the comparison for the expected usage of elastic VMs. With this metric, the proposed model also has results that are equivalent to the real system behavior. The three times between requests show an overlapped confidence interval, so we can justifiably trust the model accuracy considering a 95% confidence interval.

**Figure 7:**
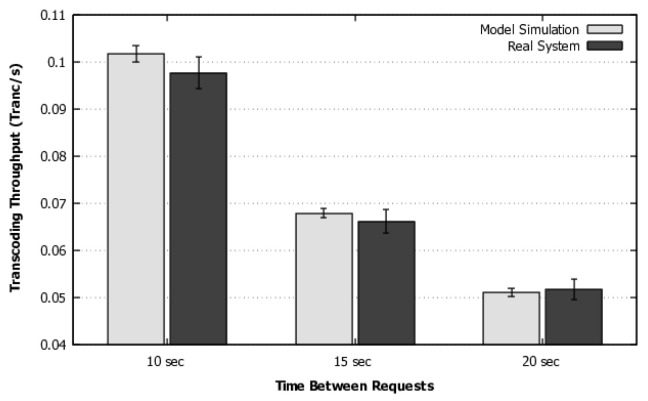
Scenario 2—Validation of throughput.

**Figure 8:**
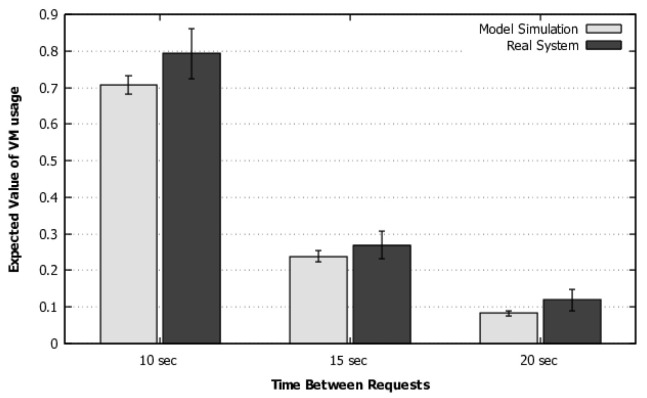
Scenario 2—Validation of elastic VM usage.

The validation of Scenario 2 was conducted with the change of the inter-arrival times for 10, 15, and 20 s. Additionally, videos were randomly selected during the execution of Jmeter. The throughput may be seen in Figure 7, and the elastic VMs usage in Figure 8. This second scenario also executed a total of 9.000 conversions. As the values from the model simulation are within the confidence intervals of the respective system measurements, we can also say that the model represents the system with 95% confidence.

### 5.2. A Case-Study

This section presents a case study that demonstrates an application of the proposed model. This study explores a space of feasible solutions for public cloud configuration considering the video transcoding system aiming to identify an optimized configuration. We employed the same transcoding times as those employed in Scenario 2 of model validation and an interval of 10 s between requests (exponentially distributed). We use the model optimization process to identify the setting that should be used in the cloud and assess the impact of three different response time constraints on the cost presented in Table 8.

The solution found by this metaheuristic should give us the parameter values for system configuration from the definition of a finite set of possible values to be analyzed for each parameter. For the parameters N_VMS, THR_SCALING_UP, THR_SCALING_DOWN, N_WORKERS), and a number of reserved instances, we will investigate the range between 1 and 10.

**Figure 9:**
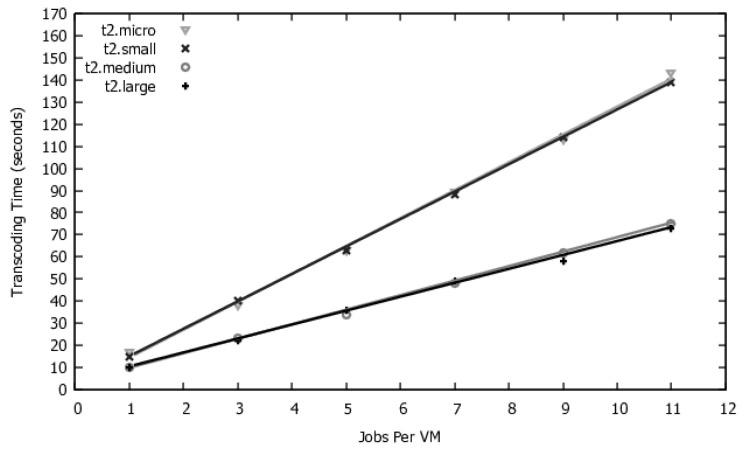
Service time for each value of N_WORKERS.

We will evaluate four VM types. The costs for each type of contract are the same as used in [43]. However, the service times for each type of VM will vary depending on the number of simultaneous transcoders each VM (**N_WORKERS**) will hold. Using the system described in Section 5.1, we measured the average service time for six distinct values of **N_WORKERS**: 1, 3, 5, 7, 9, and 11. The values found in this experiment are displayed in the points of Figure 9.

We applied a linear regression with the values found in the experiments. This enabled us to estimate service time values when N_WORKERS equals 2, 4, 6, 8, and 10. The regression function was used in the GRASP mechanism to avoid measuring each service time corresponding to a specific N_WORKERS value; we obtained the coefficient of determination $R^{2}$ above 0.99, which allows us to interpolate techniques these values with safety. Besides the service time, the choice of a VM type also affects VM instantiation time.

Table 9 shows the instantiation time for the four VM types used in this study.

**Figure 10:**
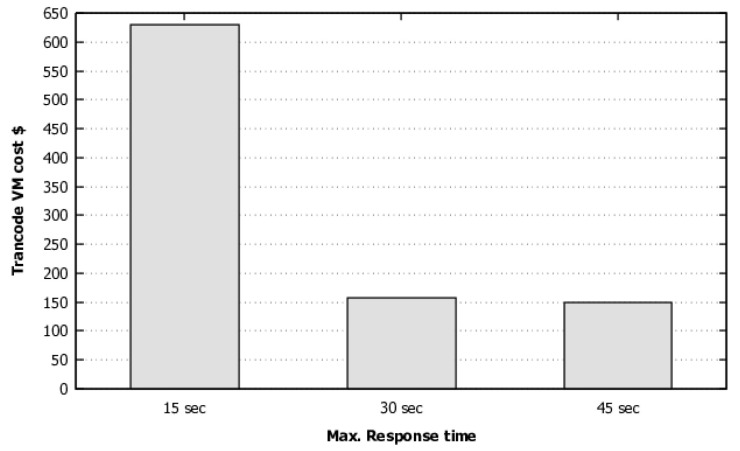
SLA cost.

The cost evaluations took into account one year of system usage. For the metrics computation, we used the equations presented in Section 4.1. Figure 10 depicts the cost of the best solution found by GRASP for each SLA. Figure 10 shows that the lower the response time, the greater will cost. However, the variation of the same SLA time interval (15 s) does not present the same cost variation, as it can also be seen that by decreasing the SLA from 30 s to 15 s, the cost increases 3.99 times. From 45 s to 30 s, a similar SLA decrease raises the cost only 1.06 times the cost.

Table 10 is another output of the optimization algorithm. It presents the configuration to be used by the system administrator in order to reach the lowest cost for each SLA, shown in Figure 10. For example, to get the lowest cost for 45 s SLA, the system configuration should be set to t2.micro VM type, employing one reserved instance, a threshold for scaling up of 5, and a threshold for scaling down VMs of 1. In addition, the number of simultaneous works per VM should be defined as 1, and the number of VMs created by scaling up for elastic VMs should be 1.

Table 11 shows the metrics for each solution. For the 45 s SLA, the cost is USD 148.74 (per year), and the average response time of that solution is 43.52 s. This system configuration also enables the conversion of 0.099 videos per second, and its usage of elastic VMs is 0.61. This result evidences that the single reserved instance is insufficient for attending the workload at all times; therefore, elastic VMs will be needed for peak periods in this case.

## 6. Conclusions

This paper proposes an SPN model capable of forecasting the behavior of auto-scaling mechanisms in a cloud computing environment. The model enables system designers to compute the trade-off between performance and cost. Furthermore, the model was validated against a real word test-bed with 95% (tested with 18,000 samples) confidence for simulation considering two distinct usage scenarios in cloud computing. Two case studies were investigated, with homogeneous and heterogeneous video streaming sizes. In both scenarios, the model was validated with the experiment results. We validated our model for a solution through numerical analysis, so it is not restricted to simulation. We employed the model with an optimization algorithm to identify how the system administrator must contract the cloud VMs and configure auto-scaling parameters to minimize the cost of each case. Finally, we presented a case study where three different mean response time constraints were defined as SLAs. The proposed model enabled us to observe that an increase in the SLA of 15 s can increase the cost by 3.99 or 1.06 times, highlighting the importance of this methodology. Future work might consider sensitivity analysis to find out the most influential elements for the metrics considered here. Operational costs for the cloud and other similar aspects might also be considered in other studies. Moreover, other optimization methods or adjustments to the GRASP method could also be assessed to reduce the time for finding good solutions for system configuration under distinct conditions.

## Figures and Tables

**Table 1:** Related work comparison.

Related Work	Measured Metrics	Architecture Planning	Formal Optimization	VM Types	Contract Types
[42]	Response time, CPU utilization	No	No	No	No
[45]	Response time, concurrent users	No	No	No	Yes
[39]	Response time, cost	No	Yes	No	Yes
[49]	Response time	No	Yes	No	No
[48]	Throughput	No	No	No	No
[47]	Response time, cost	Yes	No	No	No
[44]	Response time, cost	Yes	No	No	Yes
[46]	Response time, cost	Yes	No	Yes	No
This Work	Response time, throughput and cost	Yes	Yes	Yes	Yes

**Table 2:** Configuration parameters.

Label	Description
SCALING_UP_STRATEGY	Condition for instantiating on-demand VMs.
SCALING_DOWN_STRATEGY	Condition to destroy on-demand VMs.
THR_SCALING_UP	Instantiation threshold of on-demand VMs.
THR_SCALING_DOWN	Destruction threshold of on-demand VM.
N_VMS	Number of VMs required for each *scaling up*.
N_WORKERS	Maximum number of simultaneous jobs per VM.
$VM_{R}$	Number of reserved VMs.
SERVICE_TIME	Service Time.
INSTANTIATION_TIME	Time to instantiate a new VM.

**Table 3:** Transition attributes.

Transition	Type	Server Semantic	Weight	Priority
ARRIVAL	Timed	Single Server	-	-
SERVICE_TIME	Timed	Infinite Server	-	-
INSTATIATION_TIME	Timed	Infinite Server	-	-
T1	Immediate	-	1	1
T2	Immediate	-	1	1
T3	Immediate	-	1	2
T4	Immediate	-	1	1
T5	Immediate	-	1	1

**Table 4:** System parameters.

Label	Description
ARRIVAL	Time between request arrival.
WAIT_QUEUE	Wait for system queue availability.
WAIT	Jbs waiting to be processed.
QUEUE_SIZE	Maximum queue capacity of the system
QUEUE_CAPACITY	Capacity available in the queue.
AVL_R	Allocate processing capacity available.
PROCESSING	Jobs being processed.
OD_ALV	Available capacity of on-demand VMs.
VM_TO_Inst	Number of VMs being instantiated.
VM_OD	Maximum Number of on-demand VMs.

**Table 5:** Metrics—Mercury.

Equation	Mercury Sintax
(3)	$TP_{Exponential}=E\left\{\#PROCESSING\right\}\times \frac{1.0}{SERVICE\_TIME}$
(4)	$TP_{Erlang}=E\left\{\#PROCESSING\right\}\times \frac{1.0}{TSERVICE\_TIME\times KSERVICE\_TIME}$
(5)	$M_{RT}=\frac{(E\{\#WAIT\}+E\{\#PROCESSING\})\times ARRIVAL}{1-P\{(\#WAIT\_QUEUE=1)AND(\#QUEUE\_CAPACITY=0)AND(\#AVL\_R=0)\}}$
(6)	$EU=VM\_OD_{initial}-E\left\{\#OD_{AVL}\right\}$

**Table 6:** Transcoding time.

Scenario	Mean (s)	Distribution	Phases	Rate
Scenario 1	22.34	Erlang	350	0.0638
Scenario 2	15.58	Erlang	9	1.6843

**Table 7:** Instantiation time.

Scenario	Mean (s)	Distribution	Phases	Rate
Scenarios 1 and 2	21.14	Erlang	522	0.040

**Table 8:** Constraints—SLA.

Case	Measure	Value
1	Min. Throughput	0.099 transcoding/s
	Max. Response time	15 s
2	Min. Throughput	0.099 transcoding/s
	Max. Response time	30 s
3	Min. Throughput	0.099 transcoding/s
	Max. Response time	45 s

**Table 9:** Instantiate time.

VM Type	Time (s)
I	21.76
II	21.14
III	20.48
IV	20.36

**Table 10:** Solution—system configuration.

SLA	Parameter	Value
15 s	Vm type	t2.medium
	reserved instances	2
	THR_SCALING_UP	10.0
	THR_SCALING_DOWN	9.0
	N_WORKERS	1
	N_VMS	1
30 s	Vm type	t2.micro
	reserved instances	2
	THR_SCALING_UP	10
	THR_SCALING_DOWN	8
	N_WORKERS	1
	N_VMS	1
45 s	Vm type	t2.micro
	reserved instances	1
	THR_SCALING_UP	5
	THR_SCALING_DOWN	1
	N_WORKERS	1
	N_VMS	1

**Table 11:** Solution—system metrics.

SLA	Metric	Value
15 s	Elastic VMs usage	3.71 × 10${}^{-7}$
	Throughput	0.1 jobs/s
	Mean Response Time	13.01 s
	Cost	$630.71
30 s	Elastic VMs usage	6.651 × 10${}^{-4}$
	Throughput	0.099 jobs/s
	Mean Response Time	29.99 s
	Cost	$157.75
45 s	Elastic VMs usage	0.61
	Throughput	0.099 jobs/s
	Mean Response Time	43.52 s
	Cost	$148.74

## Data Availability

Not applicable.
